# Regional ADC values of the morphologically normal canine brain

**DOI:** 10.3389/fvets.2023.1219943

**Published:** 2023-11-08

**Authors:** Lea Carisch, Blanca Lindt, Henning Richter, Francesca Del Chicca

**Affiliations:** Clinic for Diagnostic Imaging, Department of Diagnostics and Clinical Services, Vetsuisse Faculty, University of Zurich, Zurich, Switzerland

**Keywords:** magnetic resonance imaging, canine, MRI, apparent diffusion coefficient, ADC, DWI, neuroimaging

## Abstract

**Introduction:**

Diffusion-weighted magnetic resonance imaging is increasingly available for investigation of canine brain diseases. Apparent diffusion coefficient (ADC) of normal canine brains is reported only in small numbers of subjects. The aim of the study was to investigate the ADC of different anatomical regions in the morphologically normal brain in a large population of canine patients in clinical setting. Additionally, possible influence on the ADC value of patient-related factors like sex, age and body weight, difference between the left and right side of the cerebral hemispheres, and between gray and white matter were investigated.

**Methods:**

Brain magnetic resonance studies including diffusion-weighted images of dogs presented at the Vetsuisse Faculty-University Zurich between 2015 and 2020 were reviewed retrospectively. Only morphologically normal brain magnetic resonance studies of dogs presented with neurological signs or non-neurological signs were included. Apparent diffusion coefficient values of 12 regions of interest (ROIs) in each hemisphere and an additional region in the cerebellar vermis were examined in each dog.

**Results:**

A total of 321 dogs (including 247 dogs with neurological signs and 62 dogs with non-neurological signs) of various breeds, sex and age were included. Apparent diffusion coefficient significantly varied among most anatomical brain regions. A significantly higher ADC was measured in the gray [median 0.79 (range 0.69–0.90) × 10^−3^ mm^2^/s] compared to the white matter [median 0.70 (range 0.63–0.85) × 10^−3^ mm^2^/s]. No significant differences were found between the left and right cerebral hemispheres in most of the regions, neither between sexes, different reproductive status, and not consistently between body weight groups. Age was correlated first with a decrease from dogs <1 year of age to middle-age (⩾3 to <8 years) dogs and later with an increase of ADC values in dogs ⩾8 years.

**Discussion:**

Apparent diffusion coefficient values of 25 ROIs were described in 321 morphologically normal canine brains in clinical setting. Apparent diffusion coefficient differences depending on the brain anatomical region are present. Apparent diffusion coefficient differences among age classes are present, likely consistent with brain maturation and aging. The described data can be a reference for future studies in clinical settings on the canine brain.

## Introduction

Diffusion-weighted imaging (DWI) is gaining popularity in advanced imaging in veterinary medicine and allows quantification of water molecule movement in tissue based on Brownian motion (1). Two diffusion sensitizing gradients – a dephasing and a rephasing gradient - of the same strength, are used next to a 180° radiofrequency pulse. Missing signal return is the result of moving water molecules not being rephased, causing a quantifiable reduction of the magnetic resonance (MR) signal. Movement of water molecules is anti-proportional to tissue cellularity, therefore signal loss can be used to evaluate water molecule’s degree of restricted diffusion (2). The strength of the diffusion sensitizing gradient is defined by its *b*-value (s/mm^2^).

Hence, DWI provides information about integrity and to some extent functionality of tissue, and consequently potential pathology (3–5). Based on DWI sequences, apparent diffusion coefficient (ADC) maps can be generated, and the ADC quantified (in mm^2^/s) in specific regions of interest (ROIs). Variations in ADC can be used to detect and characterize pathological processes (6).

In human medicine DWI is widely used not only with diagnostic purposes to evaluate infarction (7–10), trauma (11) and infection (12) but also for assessment of different neoplasia and individual treatment response (13–16). Further studies investigate mapping of brain regions for identification of epileptogenic zones (17, 18). Moreover, DWI has been used to describe brain maturation and aging in the human brain (19–23) and ADC is known to vary in different regions of the human brain (24). In veterinary medicine, earlier studies mainly describe the use of DWI investigating cerebrovascular incidents in dogs (25, 26), cats (27), rats (28) and brain edema in cats (29). Potential use of DWI also includes evaluation of neoplastic, inflammatory, and epileptic disorders (30–36). Studies evaluating ADC values in the morphologically normal canine brain are rare, (4, 5, 37) with reported differences depending on the anatomical region and on the side of the cerebral hemisphere (4, 5) in small numbers of dogs.

The present study aimed to investigate regional ADC in selected brain regions in the morphologically normal dog brain in clinical setting. No influence of sex, reproductive status and body weight (BW) on ADC has been hypothesized. Difference in ADC depending on the side of the brain hemisphere, on age of the patients, between gray and white matter, and among anatomical regions have been hypothesized.

## Materials and methods

### Animals

Clinical records of the small animal clinic, Vetsuisse Faculty of the University of Zurich, were reviewed retrospectively. Dogs that underwent magnetic resonance imaging (MRI) examination of the brain, including DWI sequences (see MRI technique), between January 2015 and February 2020 were reviewed. Only dogs with a morphologically normal brain MR examination (absence of abnormalities in morphology and signal intensity in all provided sequences) were included in the study. The diagnosis of a morphologically normal brain study had been stated by a board-certified radiologist, and the images were reviewed by another board-certified radiologist (FDC) at the time of inclusion in the study. If cerebrospinal fluid (CSF) tap was performed, only dogs with normal CSF tap were included. Mildly blood-contaminated samples that were marked by our laboratory, but considered diagnostic, were also included. The analysis consisted of: cell count (reference value: <5 cells/μL), protein concentration (reference value: ≤30 mg/dL) and cytological analysis of cell type as well as serological analysis if indicated. Dogs with any laboratory records of CSF tap abnormalities were excluded. As of the retrospective nature of this study, there was no need to inquire ethical consent. However, a consent form for the use of the dogs’ data for academic purposes was signed by the owners of all dogs included.

### Magnetic resonance imaging technique

The MRI examinations of all dogs were performed at the Vetsuisse Faculty, University of Zurich. A 3 T MRI scanner (Philips Ingenia 3.0 T scanner, Philips AG Healthcare, Zurich, Switzerland) and a 32-channel coil (dStream HeadNeck, 32ch MR coil; Philips AG Healthcare) or 16-channel coil (dStream HandWrist, 16ch MR coil; Philips AG Healthcare) for small dogs was used. The dogs were positioned in dorsal recumbency under general anesthesia, using case-by-case anesthetic protocols. Images of the brain including the olfactory lobes to at least the second cervical vertebrae were acquired. The standard brain protocol included the sequences with the parameters listed in Supplementary Table S1. Diffusion-weighted (DW) images were acquired in a transverse plane, setting the two *b*-values at 0 s/mm^2^ and 1,000 s/mm^2^, respectively, and using sensitivity encoding (SENSE) technique and diffusion gradients in all three planes (*x*-, *y*- and *z*-plane). Contrast medium was injected after the DW sequence (DOTAREM 0.2 mL/kg IV [Gadoteric acid; Guerbet Gmbh, Sulzbach, Germany]) followed by saline solution (NaCl 0.9% 5 mL IV).

### Data analysis

The signalment, including breed, sex, reproductive status, age, BW, and the symptomatology at presentation was recorded from the medical records of all dogs. Further, body temperature as well as duration of the MRI examination was recorded. All acquired MR images were assessed using a dedicated software (Philips IntelliSpace Portal version 10.1.1; Philips AG, Amsterdam, NL). After acquisition of the DW images, using the IntelliSpace software diffusion package, if necessary, motion correction has been performed. From the DWI sequence, the B0-treshold was adjusted to exclude background pixels from the functional map calculations. The used b-values were selected, and a new imaging series (ADC iso map) generated. Anatomical co-registration (align registration) with the T2W sequence was performed and visually controlled to ensure quality of registration prior to ROI placement.

**Figure 1:**
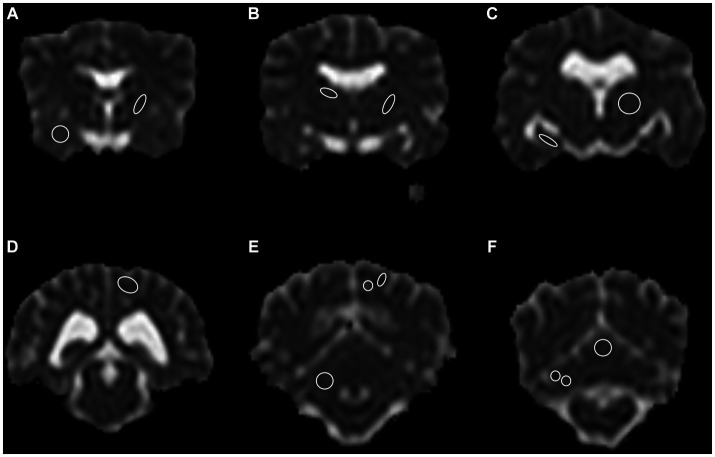
A total of 13 anatomical regions were defined as regions of interest (ROI) and drawn on both cerebral hemispheres in the transverse plane, except for the cerebellar vermis, only drawn in the center. **(A)** Right piriform lobe; left rostral internal capsule, **(B)** Right caudate nucleus; left caudal internal capsule, **(C)** Right hippocampus; left thalamus, **(D)** Left occipital lobe, gray and white matter combined, **(E)** Right cerebellum gray and white matter combined; left occipital lobe, gray and white matter separate, **(F)** Right cerebellar hemisphere gray and white matter separate; central vermis, gray and white matter combined. Note the limited spatial resolution of the apparent diffusion coefficient (ADC) map. The left side is on the right side of the image. Border ROI thickness has been increased for representative purposes. Only one side per ROI for representative purposes depicted.

A total of 13 anatomical brain regions were defined as ROIs and manually drawn in the transverse plane on the ADC map as follows: caudate nucleus, internal capsule (two locations, one rostral and one caudal), piriform lobe, thalamus, hippocampus, occipital lobe (white and gray matter combined and separate), cerebellar lobe (white and gray matter combined and separate), cerebellar vermis (Figure 1). With exception of the cerebellar vermis each ROI was drawn in both left and right hemisphere of the brain, leading to a total of 25 ROIs drawn in each dog. Each ROI was drawn on the slice representing the structure most accurately. Whenever a structure was visible on more than one slice (e.g., thalamus), the slice representing the region with the subjectively largest extent was chosen. Images of all the other sequences and planes and co-registration were used as reference to better identify the anatomy while drawing the ROI. Typically, the ROIs were drawn as follows: on the head of the caudate nucleus; on the rostral and caudal internal capsule (rostral at the level of the head of the caudate nucleus or one slice caudally; and caudal, one or two slices apart from the rostral localization); on the thalamus hemisphere as published in cats (38). The other ROIs were drawn at the level of the best definition and largest extent of the corresponding anatomical structure. ROIs were drawn excluding adjacent brain regions or CSF. ROIs were not drawn in the presence of any kind of artifacts. Considering the different anatomical structures and dogs’ sizes, the position and size of the ROI was adapted to each animal. Therefore, the size of the ROI varied strongly among the different brain regions and among dogs. Drawn regions of interest underwent an internal alignment control: in the first 20 cases, all ROIs were drawn by consensus of two of the authors (BL, a veterinarian specifically trained and FDC, an experienced board-certified radiologist). ROIs of the remaining cases were drawn by only one of the authors (BL). In case of uncertainty, the ROIs were placed by consensus of the two authors. ROI ADC values were expressed in 10^−3^ mm^2^/s and size in mm^2^.

To assess possible influence of age on ADC values, the study population was grouped in four age classes selected to match different dog life stages: <1 year – early and late puppyhood; ⩾1 to <3 years – young adults; ⩾3 to <8 years – mature adults; and ⩾8 years – senior dogs. Depending on BW, animals were grouped into three BW groups: ⩽10 kg, >10 – ⩽25 kg, >25 kg. Dogs were grouped depending on the reason for MR examination at presentation. Dogs presented with neurological signs were compared to dogs presented with non-neurological signs. Dogs with unclear neurological status were excluded for this analysis. Classifications were based on medical records.

### Statistical analysis

Data were collected in a spreadsheet software (Microsoft Excel version 16.45; Microsoft, Redmond, WA, United States) and analyzed using statistical software (IBM SPSS Statistics version 27.0; SPSS, IBM Corp, Armonk, NY, United States). Data for ADC values were not normally distributed and analyzed as non-parametrical. Descriptive statistic was performed, data reported as median (range) and mean ± SD was added when appropriate. The ADC values were tested for statistical difference according to sex and reproductive status, BW group and age classes; as well as differences between right and left cerebral hemisphere and between white and gray matter regions. The ADC between ROIs was analyzed using Friedman’s test for dependent variables, followed by Bonferroni *post-hoc* correction for multiple pairwise tests. Independent variables were analyzed with the Kruskal–Wallis test (for multiple comparisons) or the Mann–Whitney test (for pairwise comparisons), followed by Bonferroni *post-hoc* correction for multiple pairwise tests. Overall, the level of significance was set at *p* < 0.05.

## Results

A total of 321 dogs met the inclusion criteria and were included in the study (Table 1). Eighty-eight different pure breeds were represented including: French Bulldog (*n* = 22), Labrador Retriever (*n* = 16), Chihuahua (*n* = 13), Pug (*n* = 9), Australian Shepherd (*n* = 8), Border Collie (*n* = 7), German Shepherd (*n* = 7), Dachshund (*n* = 6), Maltese (*n* = 6), Miniature Spitz (*n* = 6), Yorkshire Terrier (*n* = 6), Bernese Mountain Dog (*n* = 5), Golden Retriever (*n* = 5), Jack Russel Terrier (*n* = 5), Beagle (*n* = 4), Cocker Spaniel (*n* = 4), Papillon (*n* = 4), Rhodesian Ridgeback (*n* = 4). Other pure breeds were represented by less than 3 dogs per breed. Sixty-four dogs were of mixed breed and 7 of unknown breed (breed information not disclosed in patient history). Of the pure breeds 63 were brachycephalic, 145 mesocephalic and 42 dolichocephalic (39, 40). One hundred and ninety-seven dogs were male (61.4%) and 122 (38.0%) were female. Eighty-two (25.6%) were intact male dogs, 115 (35.8%) were castrated males. Thirty-five (10.9%) were intact females and 87 (27.1%) were spayed females. Two dogs were of unknown sex (0.6%). Median age was 71.8 months (range 4.1–194.4 months). Dogs were grouped depending on the age as follows: <1 year (*n* = 21); ⩾1 to <3 years (*n* = 41); ⩾3 to <8 years (*n* = 142); and ⩾8 years (*n* = 117). BW was recorded for 320 dogs (median 16.7 kg and range 1.4–72 kg) and grouped as follows: ⩽10 kg (*n* = 104), >10 – ⩽25 kg (*n* = 109), >25 kg (*n* = 107). In one dog the BW was unknown.

**Table 1:** Demographic table of dogs included in the study.

Gender	Nr. of dogs	Age (months) median (range)	Weight (kg) median (range)
male intact	82	56.5 (4.4–155.1)	18 (2.6–56.0)
male castrated	115	77.1 (8.7–164)	16.7 (2.0–72.0)
female intact	35	52.0 (4.1–156.2)	7.3 (1.4–33.0)
female spayed	87	100.6 (14.2–194.4)	18.4 (2.1–41.7)
unknown	2	70.4 (38.1–102.8)	7.6 (7.2–7.9)
Breed			
French Bulldog	22	47.2 (11.0–119.1)	12.1 (8.0–27.2)
Labrador Retriever	16	73.1 (35.9–156.2)	30.7 (25.0–41.0)
Chihuahua	13	97.5 (40.9–142.4)	2.7 (2.0–5.2)
Pug	9	96.8 (12.9–151.7)	7.3 (6.0–9.3)
Mixed	64	82.2 (5.2–176.3)	19 (3.1–45.8)
others	197	69.5 (4.1–194.4)	18.3 (1.4–72)
Skull shape			
Brachycephalic	63	72.8 (4.1–151.7)	10.0 (2.0–38.0)
Mesocephalic	145	71 (4.2–194.4)	18.4 (1.4–56.0)
Dolichocephalic	42	69 (9.4–169.3)	24 (4.3–72.0)

The study population included clinical patients and consisted of dogs with neurological signs (*n* = 247) and with clinical signs other than neurological signs (*n* = 62). Dogs were presented for MRI examination with neurological signs (*n* = 247) including seizures (epileptic *n* = 114, non-epileptic *n* = 15), cranial nerve dysfunction (*n* = 25) vestibular dysfunction (peripheral *n* = 33, indistinguishable *n* = 6, central *n* = 1), balance impairment and gait disturbances other than vestibular dysfunction (*n* = 39), and impaired consciousness (*n* = 14). Sixty-two dogs underwent MRI examination for reasons other than neurological signs, including behavioral changes (*n* = 27), ophthalmologic disease (*n* = 13), head/neck pain (*n* = 11), ear disease without neurological deficits (*n* = 6), musculoskeletal disorder (*n* = 2), endocrine disease (*n* = 2) and one dog with nasal discharge. In 12 dogs the neurological status remained unclear, and they were excluded for the analysis of comparison between dogs with neurological signs and those presented for non-neurological signs. In 225 dogs, CSF was tapped from atlanto-occipital (*n* = 224) or lumbar site (*n* = 1). Body temperature was recorded in 253/321 dogs at the time of induction of general anesthesia (median 38.2°C; range 34.5–39.8°C) and at the end of the MR examination (median 37.0°C; range 33.5–40.5°C). The median examination time was 48 min (range 23–121 min). Typically, the DW sequence was performed in the second half of the examination. A total of 7,921 ROIs were drawn. The ADC values (mean ± SD and median and range) and the ROIs size are reported in Table 2. Artifacts prevented the drawing of one or more ROIs in 11 dogs (for a total of 100 ROIs).

**Table 2:** Apparent diffusion coefficient (ADC) and the region of interest (ROI) size in 321 dogs of the right and left hemisphere separately and averaged.

Location	Hemisphere (Nr. of ROI)	Mean ADC mean ± SD	Median	Min.	Max.	ADC hemispheres averaged median (range) mean ± SD	ROI size (mm^2^) median (range)
Caudate Nucleus	Right (321)	0.7619 ± 0.0489	0.77	0.55	0.91	0.76 (0.55–0.88) 0.7546 ± 0.05	13.4 (6.8–24.9)
	Left (321)	0.7473 ± 0.0502	0.75	0.56	0.86		
Internal Capsule rostral	Right (320)	0.7072 ± 0.0424	0.71	0.56	0.88	0.71 (0.61–0.89) 0.7068 ± 0.0412	12.8 (4.6–24.7)
	Left (321)	0.7065 ± 0.0401	0.70	0.56	0.91		
Internal Capsule caudal	Right (321)	0.7155 ± 0.0422	0.72	0.59	0.88	0.72 (0.60–0.90) 0.71360 ± 0.0407	15.5 (5.9–31.7)
	Left (321)	0.7117 ± 0.0392	0.71	0.60	0.91		
Piriform Lobe	Right (320)	0.8353 ± 0.0386	0.83	0.68	0.96	0.84 (0.70–0.97) 0.8371 ± 0.0384	18.4 (9.0–37.0)
	Left (320)	0.8390 ± 0.0382	0.83	0.71	0.98		
Thalamus	Right (320)	0.7325 ± 0.0309	0.73	0.63	0.82	0.73 (0.66–0.82) 0.7315 ± 0.0321	19.9 (9.5–37.4)
	Left (320)	0.7305 ± 0.0332	0.73	0.62	0.81		
Hippocampus	Right (318)	0.8202 ± 0.0577	0.82	0.62	0.98	0.82 (0.63–0.96) 0.8160 ± 0.0614	10.5 (4.3–17.5)
	Left (318)	0.8117 ± 0.0647	0.81	0.63	0.97		
Occipital Lobe gray & white matter av.	Right (316)	0.7202 ± 0.0535	0.72	0.59	0.89	0.72 (0.59–0.88) 0.7213 ± 0.0521	17.5 (8.5–32.4)
	Left (317)	0.7224 ± 0.0507	0.72	0.58	0.89		
Occipital Lobe gray matter	Right (314)	0.8228 ± 0.0870	0.83	0.59	1.08	0.83 (0.61–1.06) 0.8262 ± 0.0838	10.2 (4.9–19.7)
	Left (315)	0.8296 ± 0.0805	0.83	0.57	1.06		
Occipital Lobe white matter	Right (314)	0.7063 ± 0.0558	0.70	0.59	0.94	0.71 (0.62–0.87) 0.7081 ± 0.0514	8.1 (3.9–14.7)
	Left (315)	0.7098 ± 0.0467	0.71	0.57	0.86		
Cerebellum gray & white matter av.	Right (314)	0.6951 ± 0.0461	0.69	0.60	0.86	0.69 (0.59–0.83) 0.6967 ± 0.0449	16 (7.6–31.6)
	Left (313)	0.6983 ± 0.0437	0.70	0.57	0.87		
Cerebellum gray matter	Right (313)	0.7299 ± 0.0647	0.73	0.56	0.97	0.73 (0.61–0.96) 0.7343 ± 0.0615	12.3 (5.7–23.0)
	Left (312)	0.7387 ± 0.0582	0.73	0.62	0.95		
Cerebellum white matter	Right (312)	0.6855 ± 0.0529	0.69	0.55	0.87	0.69 (0.57–0.82) 0.6862 ± 0.0506	11.8 (5.7–23.0)
	Left (311)	0.6869 ± 0.0482	0.68	0.56	0.81		
Vermis gray & white matter av.	Middle (314)	0.7196 ± 0.0772	0.71	0.57	1.08	0.71 (0.57–1.08) 0.7196 ± 0.0772	9.8 (4.8–18.3)

ADC values are in (× 10^−3^ mm^2^/s). Max., maximum ADC; Min., minimum ADC; av., averaged.

**Figure 2:**
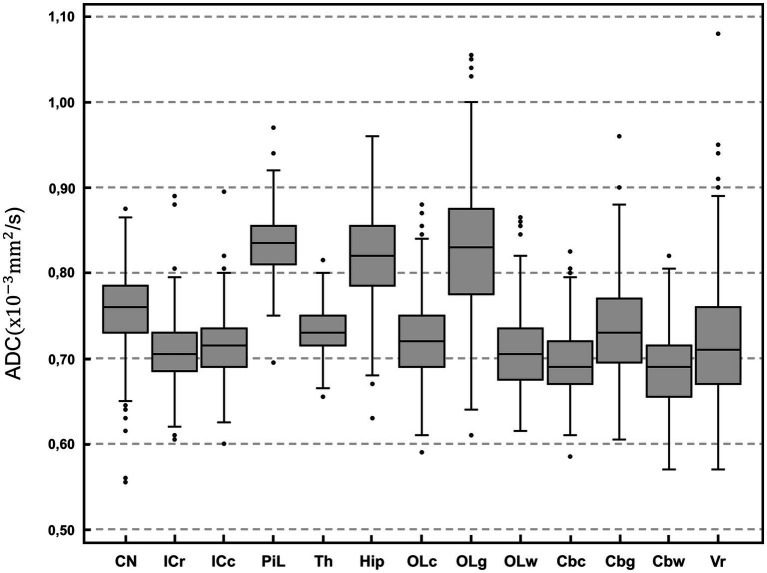
Box-plot comparison of apparent diffusion coefficient (ADC; × 10^−3^ mm^2^/s) among anatomical regions of interest. For each plot, the box represents the 25th to 75th percentiles, the horizontal line represents the median. Whiskers indicate the highest value within 1.5-times the interquartile range (IQR) and the lowest value within 1.5-times the IQR. Dots represent the outliers. CN, caudate nucleus; ICr, internal capsule rostral; ICc, internal capsule caudal; PiL, piriform lobe; Th, thalamus; Hip, hippocampus; OLc, Occipital lobe combined; OLg, occipital lobe gray matter; OLw, occipital lobe white matter; Cbc, cerebellum combined; Cbg, Cerebellum gray matter; Cbw, cerebellum white matter; Vr, vermis. Except for vermis, right and left hemisphere values are averaged.

The highest ADC values (details in Table 2 and overview in Figure 2) were recorded in the piriform lobe [0.84 (0.70–0.97) × 10^−3^ mm^2^/s], followed by the occipital lobe gray matter [0.83 (0.61–1.06) × 10^−3^ mm^2^/s] and the hippocampus [0.82 (0.63–0.96) × 10^−3^ mm^2^/s]. The lowest ADC value was recorded in the cerebellar white matter region [0.69 (0.57–0.82) × 10^−3^ mm^2^/s]. The largest ROI size was drawn in the thalamus [19.9 (9.5–37.4) mm^2^] and the smallest in the white matter of the occipital lobe [8.1 (3.9–14.7) mm^2^; Table 2]. For most of the analyzed ROIs, the difference in ADC was statistically significant (Table 3). Significantly higher ADC (*p* < 0.001) were measured in the gray [0.79 (0.69–0.90) × 10^−3^ mm^2^/s] compared to the white matter [0.70 (0.63–0.85) × 10^−3^ mm^2^/s]. Statistically significant difference was present between right and left hemisphere in 5 ROIs (*p* < 0.001–*p* = 0.047): the caudate nucleus [left 0.75 (0.56–0.86) × 10^−3^ mm^2^/s, right 0.77 (0.55–0.91) × 10^−3^ mm^2^/s], internal capsule caudal [left 0.71 (0.60–0.91) × 10^−3^ mm^2^/s, right 0.72 (0.59–0.88) × 10^−3^ mm^2^/s], piriform lobe [left 0.83 (0.71–0.98) × 10^−3^ mm^2^/s, right 0.83 (0.68–0.96) × 10^−3^ mm^2^/s], hippocampus [left 0.81 (0.63–0.97) × 10^−3^ mm^2^/s, right 0.82 (0.62–0.98) × 10^−3^ mm^2^/s] and cerebellum gray matter [left 0.73 (0.62–0.95) × 10^−3^ mm^2^/s, right 0.73 (0.56–0.91) × 10^−3^ mm^2^/s]. No hemisphere side had consistently higher ADC values. No statistically significant difference in ADC values between male and female dogs or depending on reproductive status was present.

**Table 3:** Statistical comparison (Friedman test with *post-hoc* test Bonferroni correction for multiple testing) of ADC values among anatomical regions of interest.

	CN	ICr	ICc	PiL	Th	Hip	OLc	OLg	OLw	Cbc	Cbg	Cbw	Vr
*CN*													
*ICr*	#												
*ICc*	#	1.000											
*PiL*	#	#	#										
*Th*	#	#	#	#									
*Hip*	#	#	#	0.093	#								
*OLc*	#	0.029*	1.000	#	0.575	#							
*OLg*	#	#	#	0.024*	#	1.000	#						
*OLw*	#	1.000	1.00	#	#	#	0.131	#					
*Cbc*	#	1.000	0.007*	#	#	#	#	#	0.611				
*Cbg*	#	#	0.004*	#	1.000	#	1.000	#	#	#			
*Cbw*	#	0.008*	#	#	#	#	#	#	0.002*	1.000	#		
*Vr*	#	1.000	1.000	#	0.005*	#	1.000	#	1.000	0.001*	0.036*	#	

Values marked with * are statistically significant (*p* < 0.05) and values below 0.001 are indicated by #. CN, caudate nucleus; ICr, internal capsule rostral; ICc, internal capsule caudal; PiL, piriform lobe; Th, thalamus; Hip, hippocampus; OLc, Occipital lobe combined; OLg, occipital lobe gray matter; OLw, occipital lobe white matter; Cbc, cerebellum combined; Cbg, Cerebellum gray matter; Cbw, cerebellum white matter; Vr, vermis.

**Figure 3:**
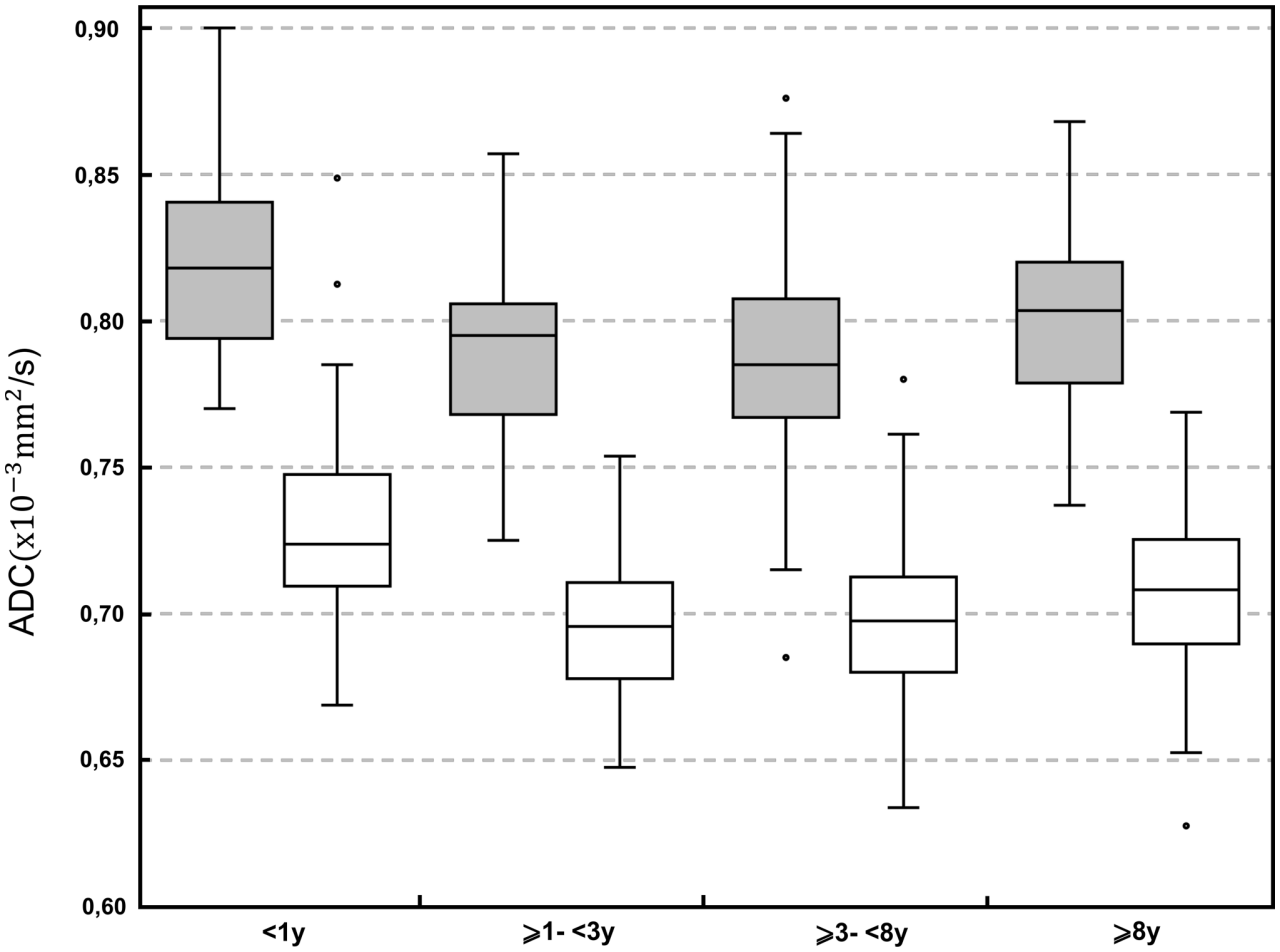
Boxplot of the apparent diffusion coefficient (ADC; × 10^−3^ mm^2^/s) in the different age classes: <1; ⩾1 to <3 years; ⩾3 to <8 years; and ⩾8 years. Grey boxes represent gray matter; white boxes represent white matter. For each plot, the box represents the 25th to 75th percentiles, the horizontal line represents the median. Whiskers represent the highest value within 1.5-times the interquartile range (IQR) and the lowest value within 1.5-times the IQR. Dots represent the outliers.

Depending on the BW classes, differences were found in 3 ROIs (occipital lobe combined matter, cerebellum gray matter, cerebellar vermis; *p* < 0.001–*p* = 0.011). In 1 ROI (occipital lobe combined matter) the median values were higher within the ⩽10 kg group whereas in 2 ROIs (vermis, cerebellum gray matter) the median values were higher within the >25 kg group. No BW group had consistently higher ADC. Statistically significant differences were mainly but not consistently found between the age class <1 year to the middle-aged class (⩾3 to <8 years) with highest median values within dogs <1 year of age (Figure 3). Overall, median ADC values within gray and white matter decreased from dogs under 1 year of age to middle-aged dogs (⩾3 to <8 years) and increased again in dogs over 8 years of age.

Statistically significant differences were found in the internal capsule (rostral and caudal) in dogs presented with neurological signs compared to dogs presented with non-neurological signs. In both ROIs the ADC values were higher in the group with neurological signs. No difference was found between ADC drawn by consensus and values from the single observer.

## Discussion

The study describes the regional ADC values in morphologically normal brains in a large population of canine patients in clinical settings. The highest ADC values were measured in the piriform lobe followed by the occipital lobe and the hippocampus. Other studies report the highest ADC values in the hippocampus followed by the piriform lobe (4); in the cortex of the frontal and parietal lobes followed by the occipital lobe and hippocampus (5). The lowest ADC value was found in the cerebellar white matter, similar to findings in cats (3). As shown in Table 4, ADC values of the present study were substantially similar as reported (5), except for the gray matter of the cerebellum. Higher regional ADC values have also been reported (4). However, different ADC values of different studies should be compared cautiously because they are strongly influenced by differences in equipment and technology used. This includes the system type, the strength of the magnet and the applied scanning protocol. Even using the same coil, variations in ADC values are reported (41). To minimize these variations, the same magnet, coils, and protocol were used in the present study. Similar ADC values were reported with identical magnet strength and *b-*values (5).

**Table 4:** Comparison of ADC values between current and previous studies.

Equipment				
		Current study	MacLellan et.al.	Hartmann et.al
MR equipment		Philips Ingenia 3.0 T scanner. Philips AG Healthcare. Zurich. Switzerland	GE Signa HDx 3.0-T MRI scanner. GE Medical Systems. Milwaukee. WI. USA	Philips Intera Gyroscan. Philips Healthcare. Hamburg. Germany
Field strength b-value		3 Tesla 0 and 1′000 s/mm^2^	3 Tesla 0 and 1′000 s/mm^2^	1 Tesla 0 and 800 s/mm^2^
Coils		dStream HeadNeck. 32ch MR coil; dStream HandWrist. 16ch MR coil	HD T/R quad extremity. Invivio, Pewaukee. Wis.	SENSE-flex M coil
Sample size		321 dogs	13 dogs	10 dogs
ADC				
Location	Hemisphere	Current study mean ± SD (× 10^−3^ mm^2^/s)	MacLellan mean ± SD (× 10^−3^ mm^2^/s)	Hartmann mean ± SD (× μm^2^/s)
Caudate Nucleus	Right	0.7619 ± 0.0489		843.3 ± 94.3
	Left	0.7473 ± 0.0502		962.4 ± 103.2
Internal Capsule rostral	Right	0.7072 ± 0.0424		
	Left	0.7065 ± 0.0401		
Internal Capsule caudal	Right	0.7155 ± 0.0422		
	Left	0.7117 ± 0.0392		
Piriform Lobe	Right	0.8353 ± 0.0386		895.0 ± 50.6
	Left	0.8390 ± 0.0382		935.9 ± 37.8
Thalamus	Right	0.7325 ± 0.0309	0.7766 ± 0.0368	792.2 ± 31.0
	Left	0.7305 ± 0.0332	0.7804 ± 0.0426	823.4 ± 30.1
Hippocampus	Right	0.8202 ± 0.0577	0.8776 ± 0.0737 (averaged)	1052.9 ± 109.7
	Left	0.8117 ± 0.0647		1035.7 ± 109.0
Occipital Lobe gray & white matter averaged	Right	0.7202 ± 0.0535	0.7982 ± 0.0363	
	Left	0.7224 ± 0.0507	0.7885 ± 0.0391	
Occipital Lobe gray matter	Right	0.8228 ± 0.0872	0.8866 ± 0.0376	
	Left	0.8296 ± 0.0805	0.8632 ± 0.0580	
Occipital Lobe white matter	Right	0.7063 ± 0.0558	0.6936 ± 0.0413	
	Left	0.7098 ± 0.0467	0.6897 ± 0.0272	
Cerebellum gray & white matter averaged	Right	0.6951 ± 0.0461	0.7196 ± 0.0573	
	Left	0.6983 ± 0.0437	0.7038 ± 0.0573	
Cerebellum gray matter	Right	0.7299 ± 0.0647	0.8369 ± 0.0654	
	Left	0.7387 ± 0.0582	0.8349 ± 0.0552	
Cerebellum white matter	Right	0.6855 ± 0.0529	0.7196 ± 0.0573	
	Left	0.6869 ± 0.0482	0.7038 ± 0.0573	

MacLellan et al. (5) and Hartmann et al. (4).

Apparent diffusion coefficient values of white matter regions were significantly lower compared to gray matter. This is expected since white matter, contrary to gray matter, mainly consists of myelinated axons, which act as diffusion barriers and lead to lower ADC values (5). Moreover, myelinated axons are responsible for a directional diffusion pattern, parallel to the axonal pathways, called anisotropy (5, 42). This finding is reproducible in dogs and humans (4, 5, 23, 43) and has been recently described also in cats (3).

The median ADC significantly differed in 5 ROIs between right and left cerebral hemisphere. Apparent diffusion coefficient was not consistently higher on one hemisphere side: significantly higher ADC was found in the right caudate nucleus, internal capsule and hippocampus, and in the left piriform lobe and cerebellum gray matter. Statistically higher ADC values in the left cerebral hemisphere have been reported (4), as well as higher, but not significantly different values in the right hemisphere (5) in a smaller number of dogs. Asymmetry in ADC values in several regions has been described in humans, with some correlation to age and gender (44). One of the most accredited explanations is that asymmetry in cerebral structures or neuronal density could influence cell density and therefore diffusivity, causing differences in ADC depending on the side. Higher neuronal density leads to lower diffusion and the right canine brain hemisphere has been reported being heavier than the left (45). Handedness is a discussed explanation for laterality in human brains (46) influencing cell density and therefore diffusivity, causing differences in ADC depending on the side (47). Pawedness in dogs and associated brain asymmetry is also described. However, in studies on dogs that do show pawedness, no overrepresentation of the left or right paw was found (48–50) This could support the lack of consistency for higher ADC values in one side of the hemispheres in our study.

Similarly, to our results, no difference in ADC values depending on the sex has been found in cats (3). Studies in canines and humans showed an effect of age on diffusivity in the brain. In humans ADC values decrease until adulthood and increase during the senescence period. Myelination during adolescence and, inversely, loss of extracellular volume and demyelination during senescence have been discussed as underlying causes. (5, 44, 51, 52) In our study, median ADC values decreased from dogs younger than 1 year of age to middle aged dogs (⩾3 to <8 years) and increased again in the age class of dogs older than 8 years. The changes in myelination would support our results, considering the different life span between humans and dogs, and likely according to different pace of age-relating changes of the brain.

Similarly, as reported (5), no consistent differences between BW groups and median ADC values were found in our study. There is no convincing explanation for the described difference in three ROIs depending on the BW. Two of these ROIs (caudal internal capsule and gray matter of the cerebellum) were among the smaller sized ROIs. It can be speculated that through limited spatial resolution in the ADC maps, further decreasing in smaller animals, ROI placement in smaller dogs may be more inaccurate than in larger animals. In smaller dogs, pixels may represent a larger proportion of the optically resolved patient anatomy and a mismatch between morphological sequences, with higher spatial resolution, might lead to inaccurate ROI placement. Therefore, differences to smaller sized dogs may simply be an inaccuracy effect.

The majority of the dogs were, as expected, presented with neurological signs (*n* = 247), less than the half of them with epileptic seizures. In dogs diagnosed with idiopathic epilepsy, the ADC values during the interictal phase are described to be higher in the piriform lobe and the semioval center, compared to values of healthy dogs. This fact might be explained by cell loss and an increased intercellular space (35). During ictus the opposite is found, and regional ADC decreases in human patients and canine experimental model of status epilepticus (53, 54). Our data did not confirm this variation in the ADC of the piriform lobe in dogs depending on the clinical signs. No ADC differences were found between dogs presented with neurological signs vs. dogs presented with non-neurological signs in all ROI, except for the internal capsule. In our patient population, dogs presented with neurological sings included highly heterogeneous etiology and the difference in ADC in the internal capsule cannot be clearly interpreted. It can be only speculated if the different ADC could suggest a possible link between neurological signs and region integrity or if this variation could represent a cause or the sequela of the neurological signs. Since a final histological diagnosis was not available in these dogs, caution should be used in the interpretation of these results.

Many other patient related factors can influence the ADC. In rats and mice, ADC is described to correlate positively with body temperature (55, 56). The patient temperature was recorded when available and was maintained in the physiological range. The exact temperature at the time of the acquisition of the DW images was not recorded though, and its influence on the ADC remains unknown. Anesthesia and its effect on neuronal activity can also influence water diffusivity. This phenomenon may cause increased ADC in specific brain regions, reflecting decreased neuronal activity (57). In our study, anesthesia was not standardized, and the anesthetic protocol elected on case-by-case evaluation by the anesthetist in charge. The possible influence of anesthesia was therefore not investigated.

The major limitation of this study is the examined population, consisting of clinical patients. Even though only dogs with morphologically normal MR brain studies were included, non-visible or microstructural pathological processes cannot be ruled out since most of the dogs were presented with neurological signs and obviously, no histopathological examination was possible. Pathologies of different nature beyond the optical resolution of the standard morphological MR images might go undetected (58, 59) but could potentially affect the ADC. Moreover, the analyzed population suffers the selection bias of a clinical population. The age groups are unequal in size, being older dogs presented more often for MRI examination. This disproportionate distribution of age group size could have had an impact on the results.

Another limitation consists in the retrospective nature of the study. Individual case management and the time between MRI examination and presence of clinical signs was neither investigated nor standardized and could influence ADC values in relation to the onset of the clinical signs. Apparent diffusion coefficient values were analyzed in the context of the clinical patient work-up. The influence of technically related factors potentially affecting the ADC, phantom reference values or signal-to-noise ratio in every single examination, as well as influence of non-optically visible artifacts were not investigated. The reported ADC based on the images acquired with described technique does not allow quantification of the anisotropy, particularly marked in the white matter of the brain. ADC ROI placement was largely performed by a single observer and no repetition of measurements was performed. Possible inter-observer variabilities should be therefore considered a limitation.

## Conclusion

The present study investigates the ADC in different regions of canine brain with morphologically normal MRI examination. Three-hundred and twenty-one dogs of varying breeds, sex, reproductive status, and age were included in the study. Apparent diffusion coefficient significantly varies among most anatomical brain regions. Apparent diffusion coefficient was significantly lower in the white than in the gray matter. The left and right cerebral hemispheres had similar ADC values in most of the regions, and no differences depending on sex or reproductive status were observed. No consistent correlation between body-weight groups and ADC values was found. Age was correlated first with a decrease and later increase of ADC values, likely consistent with brain maturation and aging. The described data can be reference for future studies in clinical settings on the canine brain.

## Data availability statement

The original contributions presented in the study are included in the article/Supplementary material, further inquiries can be directed to the corresponding author.

## Ethics statement

A consent form for the use of the dogs’ data for academic purposes was signed by the owners of all dogs included.

## Author contributions

Conception and design done by FC and LC. Acquisition of data done by BL. Statistical analysis done by HR. Drafting of the article done by LC. All authors contributed to the article and approved the submitted version.
