# Supplementary material for: Regional ADC values of the morphologically normal canine brain
Source: Front Vet Sci. 2023 Nov 8;10:1219943. doi: 10.3389/fvets.2023.1219943 (PMC10663295; doi:10.3389/fvets.2023.1219943)
Supplement: Supplementary file 1 [file Table_1.docx]

***Table S1****: Standard brain protocol parameters used. TR = repetition time; TE = echo time; FLAIR = fluid-attenuated inversion recovery; 3D = three-dimensional; T2W = T2-weighted; T1W = T1-weighted; DWI = diffusion weighted imaging*

| **Sequence** | **Planes** | **Spin echo** | **TR/TE (ms)** | **Flip angle** | **Field of view** | **Voxel size (mm)** | **Slice gap**  **(mm)** | **Slice thickness (mm)** |
| --- | --- | --- | --- | --- | --- | --- | --- | --- |
| *T2W* | transverse. dorsal. sagittal | turbo spin echo | 5031/100 | 90° | adapted to animal | 0.4x0.53x2.8 | 0.28 | 2.8 |
| *FLAIR* | transverse | turbo spin echo | 11000/125 | 90° | adapted to animal | 0.43x0.56x2.80 | 0.28 | 2.8 |
| *T1W*  *pre-/postcontrast (3D)* | transverse | turbo fast echo | 11/5.2 | 8° | adapted to animal | 0.70x0.70x0.70 | 0 | 0.7 |
| *DWI* | transverse | - | 3750/95 | 90° | adapted to animal | 1.6x2.03x2.00 | 1 | 2 |
